# Supplementary material for: Automated 1D Helmholtz coil design for cell biology: Weak magnetic fields alter cytoskeleton dynamics
Source: PLoS One. 2025 Aug 5;20(8):e0321133. doi: 10.1371/journal.pone.0321133 (PMC12324680; doi:10.1371/journal.pone.0321133)
Supplement: S1 File — (PDF) [file pone.0321133.s001.pdf]

Aug 25, 2024

Coil construction

DOI
dx.doi.org/10.17504/protocols.io.n2bvjneewgk5/v1

Clarice D. Aiello[1]

[1]Quantum Biology Tech (QuBiT) Lab

Quantum Biology Tech (Q…

Clarice D. Aiello
Quantum Biology Tech (QuBiT) Lab

OPEN ACCESS

DOI: **dx.doi.org/10.17504/protocols.io.n2bvjneewgk5/v1**

Protocol Citation: Clarice D. Aiello 2024. Coil construction. **protocols.io** **https://dx.doi.org/10.17504/protocols.io.n2bvjneewgk5/v1**

License: This is an open access protocol distributed under the terms of the **Creative Commons Attribution License**, which permits unrestricted use, distribution, and reproduction in any medium, provided the original author and source are credited

Protocol status: Working
Protocol still in use as of Aug 2024

Created: August 23, 2024

Last Modified: August 25, 2024

Protocol Integer ID: 106387

Abstract

To systematically quantify magnetic field effects in cell biology, it is crucial to produce fields that can be automatically adjusted and that are stable throughout an experiment's duration, usually operating inside an incubator. Here, we report on the design of a fully automated 1D Helmholtz coil setup that is internally water cooled, thus eliminating any confounding effects caused by temperature fluctuations. The coils also allow cells to be exposed to magnetic fields from multiple directions through automated controlled rotation.

This protocol accompanies arXiv manuscript http://arxiv.org/abs/2406.19555.

Materials

1 Here are the materials required and the assembly instructions for the coils.
* Copper wire (0.71 mm diameter);
* 3D-printed coil holder in thermoplastic material (CAD files found **here**);
* Machined coil enclosure made out of Delrin or aluminum (CAD files also found **here**);
* Thermally conductive glue (*e.g.*, 10 g of GENNEL G109);
* Two-part epoxy (*e.g.*, J-B Weld 8276);
* Silicone sealant (*e.g.* , Aqueon);
* Multi-axes magnetometer for testing (*e.g.*, F71 Multi-axis sensor).

Assembly instructions

2

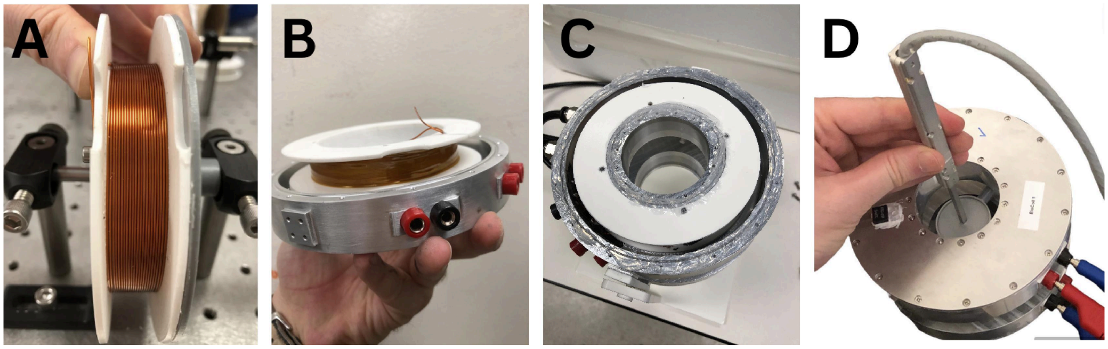

* 3D-print the holder around which the coils are wound, and machine the coil enclosure. Choose a material (*e.g.*, a thermoplastic) that is sturdy, lightweight, and corrosion-free;
* **A.** Wind the copper wire 96 times around the 3D-printed holder. Secure the coils' surfaces using the thermally conductive glue;
* **B.** Place the wound coils and their holder inside the coil enclosure and establish the necessary electrical connections. To seal the electrical connections, apply the two-part epoxy;
* **C.** Prevent water leakage by applying the silicone sealant before screwing the caps of the coil enclosure. Firmly fix the caps to the coil enclosure to ensure a watertight seal;
* **D.** Use the multi-axes magnetometer to measure the produced magnetic fields when different currents are applied.
