# Supplementary material for: Automated 1D Helmholtz coil design for cell biology: Weak magnetic fields alter cytoskeleton dynamics
Source: PLoS One. 2025 Aug 5;20(8):e0321133. doi: 10.1371/journal.pone.0321133 (PMC12324680; doi:10.1371/journal.pone.0321133)
Supplement: Supplementary Materials — (PDF) [file pone.0321133.s002.pdf]

Supplementary materials

SSI Thermal control in coil design

The rise in water temperature in a coil can be calculated using the power dissipated by it. Electric currents generate heat due to resistance in the wire, which is then transferred to the water by convection. The heat produced by the coil depends on both current and resistance. Effective transfer relies on water flow and heat capacity. The power dissipated in each coil equals the rate of internal energy increase added to the rate of energy transfer from the coil to its surroundings through its surface. In other words,

$$P_{\text{diss}} = \frac{dU}{dt} + \oint_S J \cdot E \, dA. \tag{SE1}$$

The expression P_{diss} represents the power dissipated [W], dU/dt is the rate of increase of internal energy [J/s], $\oint_S J \cdot E \, dA$ denotes the rate of energy transfer from the coil to its surroundings [J/s], J is the current density vector [A/m^2], E is the electric field vector [V/m], and dA represents an infinitesimal area element on the surface S [m^2]. Once the system reaches a stationary state [56], only the second term in Eq. SE1 persists, determining the maximum temperature increase of the coil:

$$\Delta T_{\text{max}} = \frac{(IN)^2 \rho}{4 h f_c A^{3/2}}. \tag{SE2}$$

In the above, I denotes the current [A], N represents the number of turns in the coil, ρ stands for the electrical resistivity ($\Omega \cdot$ m), h is a heat transfer constant ranging from $5 - 25\,\text{W}/(\text{m}^2 \cdot \text{K})$ [57], f_c signifies the fill factor, and A represents the total cross-sectional area of the coil [m^2]. The fill factor, representing the fraction of the coil's cross-sectional area filled with conductor material, is given by the formula $f_c = N\pi t^2/4A$, where t is the diameter of the wire. The fill factor gauges the efficiency of the conductor material in using the space within the coil. In our coil, the fill factor is 0.74, which falls within the typical range of 0.6 to 0.8. Operating the coils with a current of 1 A to generate a magnetic field of $B = 1.8$ mT results in a dissipated power of $P_{\text{diss}} = 2.74$ W. At this level of dissipated power, a flow rate of 1 L/min yields a temperature rise of only 0.4 °C in the water exiting the coil enclosure (Fig. SF1). As this heat is also radiated into the surrounding air (Eq. SE1), using a thermometer with a resolution of 0.1 °C, in practice we could not measure any temperature increase in the space between the coils, where the Petri dish is placed during experiments.

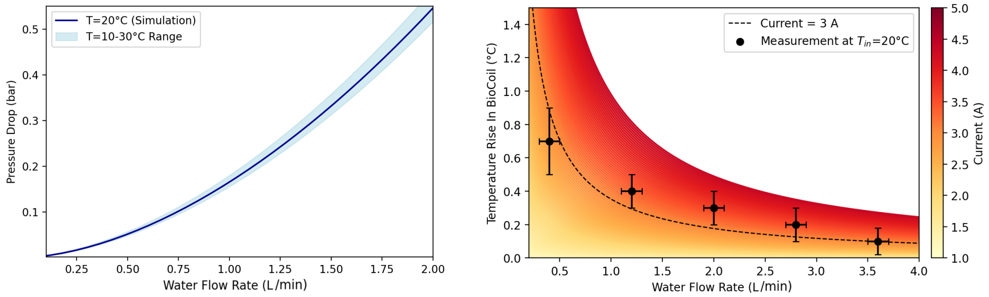

Fig. SF1. Simulated temperature rise within coils whose initial temperature is 20 °C is in agreement with measurements. Left, the simulated relationship between pressure drop and required flow rate for the 4 m long water pipes (inner cross section of 16 mm^2) connected to the coils. The used chiller pump needs to be capable of managing such a pressure drop. Right, numerical simulation of how the coils' temperature responds to varying levels of applied current up to 5 A, as a function of the water flow rate in the system. For example, at an operational current of 3 A (generating a $\sim 3 \times 1.8 \sim 5.4$ mT magnetic field), a flow rate of 2 L/min effectively keeps the temperature increase within a range of 0.4 °C for the water exiting the coil. At an operational current of 1 A, no temperature rise of 0.1 °C or above could be measured at the sample position.

To avoid system overheating, a temperature control sensor has been integrated and attached to the surface of the coil enclosure. The sensor is a passive thermostat (KSD9700), engineered to interrupt the circuit when the coil overheats. With a flow rate set at 1 L/min, the pressure drop across the entire coil configuration, starting from the point where water enters a 4 mm inner diameter pipe and continuing until it exits the enclosure, is simulated to be $\Delta P \sim 0.33$ bar. It must be noted that we considered a open flow setup and didn't take the back pressure [58] that your connected coil might feed into the system.

SSII Addressing condensation on the coils

When using water to cool the coils, it is imperative to prevent air condensation on the enclosure. We determined the lowest temperature (dew temperature) [51] under various incubator humidity levels below which air condensation occurs. Eq. SE3 estimates the dew point temperature (T_{dew}) from relative humidity (RH) and temperature (T) using empirical constants.

$$T_{\mathrm{dew}}\,(^\circ\mathrm{C}) = c \cdot \left(\log\left(\frac{RH}{100}\right) + \frac{b \cdot T}{c+T} \right) \times \left(b - \log\left(\frac{RH}{100}\right) - \frac{b \cdot T}{c+T} \right)^{-1}. \qquad \text{(SE3)}$$

The numerical constants, $a = 6.112$, $b = 17.62$, and $c = 243.12$, used in this context are sourced from [59]. Our simulations suggest that, in an incubator accurately adjusted to the standard conditions of 37 °C and 60 % humidity, the temperature of the water circulating within the coils needs to be above 28 °C in order to prevent the air condensation (Fig. SF2).

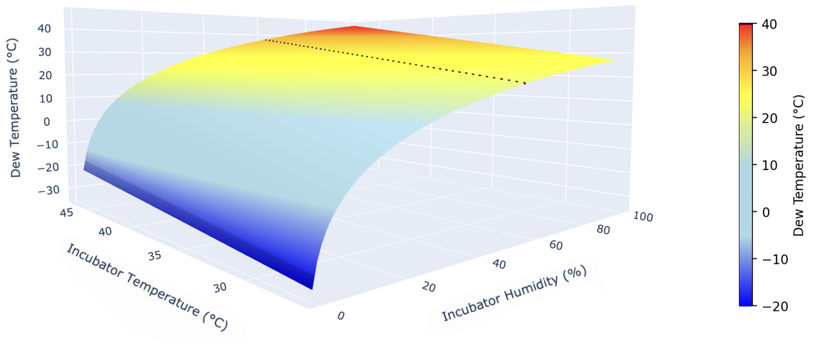

Fig. SF2. Estimating the air's dew point temperature in correlation to the temperature and humidity levels within an incubator. To prevent moist condensation on the surface of the enclosure placed inside an incubator, it is necessary to maintain the water temperature circulating within the enclosure above 28 °C. The dashed line illustrates how to adjust the water cooling temperature in the coil for various incubator temperatures while maintaining a constant incubator humidity level at 60%.

SSIII Biological replicates composing the dataset of Fig. 5B

The F-actin alignment data of Fig. 5B was obtained by analysis of 229 cells studied over the course of two biological replicates. We present below a similar plot for each one of the identical experiments.

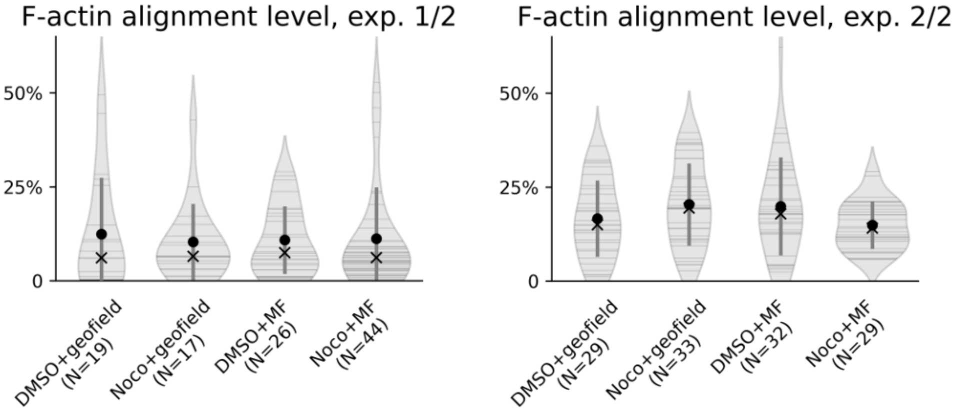

Fig. SF3. Separate data for each one of the biological replicates composing the dataset of Fig. 5B.
